# High temperature perception in leaves promotes vascular regeneration and graft formation in distant tissues

**DOI:** 10.1242/dev.200079

**Published:** 2022-02-28

**Authors:** Phanu T. Serivichyaswat, Kai Bartusch, Martina Leso, Constance Musseau, Akira Iwase, Yu Chen, Keiko Sugimoto, Marcel Quint, Charles W. Melnyk

**Affiliations:** 1 Department of Plant Biology, Swedish University of Agricultural Sciences, Ulls gränd 1, 765 51 Uppsala, Sweden; 2 Institute of Molecular Plant Biology, Department of Biology, ETH Zürich, 8092 Zürich, Switzerland; 3 Institute of Agricultural and Nutritional Sciences, Faculty of Natural Sciences III, Martin Luther University Halle-Wittenberg, Betty-Heimann-Str. 5, 06120 Halle (Saale), Germany; 4 RIKEN Center for Sustainable Resource Science, Yokohama 230-0045, Japan; 5 Department of Biological Sciences, Faculty of Science, The University of Tokyo, Tokyo 113-8654, Japan

**Keywords:** Grafting, Regeneration, Temperature sensing, Auxin transport, Vascular biology, Parasitic plants, *Arabidopsis thaliana*

## Abstract

Cellular regeneration in response to wounding is fundamental to maintain tissue integrity. Various internal factors including hormones and transcription factors mediate healing, but little is known about the role of external factors. To understand how the environment affects regeneration, we investigated the effects of temperature upon the horticulturally relevant process of plant grafting. We found that elevated temperatures accelerated vascular regeneration in *Arabidopsis thaliana* and tomato grafts. Leaves were crucial for this effect, as blocking auxin transport or mutating *PHYTOCHROME INTERACTING FACTOR 4* (*PIF4*) or *YUCCA2/5/8/9* in the cotyledons abolished the temperature enhancement. However, these perturbations did not affect grafting at ambient temperatures, and temperature enhancement of callus formation and tissue adhesion did not require *PIF4*, suggesting leaf-derived auxin specifically enhanced vascular regeneration in response to elevated temperatures. We also found that elevated temperatures accelerated the formation of inter-plant vascular connections between the parasitic plant *Phtheirospermum japonicum* and host *Arabidopsis*, and this effect required shoot-derived auxin from the parasite. Taken together, our results identify a pathway whereby local temperature perception mediates long distance auxin signaling to modify regeneration, grafting and parasitism.

This article has an associated ‘The people behind the papers’ interview.

## Abstract (precis)

**Summary:** This paper describes a pathway in which high temperatures are perceived by the leaves to promote an auxin-dependent signal that accelerates vascular regeneration, graft formation and plant parasitism in distant tissues.

## INTRODUCTION

Various abiotic and biotic stresses including temperature extremes, herbivory and cutting induce damage that needs to be repaired (Ikeuchi et al., 2019). These stresses are a source of tissue damage, but the environment can also promote regeneration. One notable example is the influence of temperature upon regeneration. Elevated temperatures enhance the formation of stem-cell like tissues, termed callus, that aid the wound healing process (Lee and Seo, 2017). Increased temperatures also improve the horticultural process of plant grafting (Bartusch et al., 2020; Shibuya et al., 2008), which consists of cutting and joining different shoots, known as scions, and roots, known as rootstocks, together to improve stress tolerance and yields (Melnyk and Meyerowitz, 2015; Mudge et al., 2009). At the cut sites, grafts initially form callus (Ikeuchi et al., 2017) that seal the wound, followed by vascular division and differentiation that allows phloem and xylem reconnection (Melnyk et al., 2015). Related processes occur during other forms of wound healing such as when callus forms at the site of cutting or cell layers divide and differentiate to restore tissue integrity after cell ablation (Iwase et al., 2011; Marhava et al., 2019). A common theme to regeneration in plants is the involvement of auxin. Auxin is mainly produced in young leaves (Ljung et al., 2001) and accumulates at the site of injury (Canher et al., 2020) where auxin responses increase (Asahina et al., 2011; Hoermayer et al., 2020; Melnyk et al., 2015). Auxin plays an important role in regenerating the vasculature: disrupting auxin response or auxin transport inhibits graft formation (Matsuoka et al., 2016; Melnyk et al., 2015) and blocks xylem connection formation between parasitic plants and their hosts during the conceptually related process of parasitic plant infection (Ishida et al., 2016; Wakatake et al., 2020).

The success of wound healing at the graft junction depends on internal factors including hormones and the developmental stage, but also on external factors such as light intensity (Bartusch et al., 2020), photoperiod (Marsch-Martínez et al., 2013) and temperature (Turnbull et al., 2002). In *Arabidopsis*, elevated ambient temperature alters growth and developmental traits including elongating hypocotyls, petioles and roots (Quint et al., 2016). The transcription factor *PHYTOCHROME INTERACTING FACTOR 4* (*PIF4*) is the major temperature-signaling hub in aerial tissues (Delker et al., 2014; Koini et al., 2009; Lee et al., 2021). High temperatures deactivate the photoreceptor Phytochrome B (PhyB) and release its suppression of *PIF4*. The PIF4 protein directly upregulates the expression of *YUCCA8* (*YUC8*), a gene associated with auxin biosynthesis (Franklin et al., 2011; Sun et al., 2012). In *Arabidopsis*, high temperatures promote a mobile auxin signal (Bellstaedt et al., 2019) that is activated by epidermal PIF4 in cotyledons (Kim et al., 2020). Cotyledon-produced auxin is then transported via the petioles to the hypocotyl where it causes brassinosteroid-induced cell elongation (Bellstaedt et al., 2019).

Elevating temperatures during graft healing improves grafting success rates in plants including *Arabidopsis* (Bartusch et al., 2020; Turnbull et al., 2002), watermelon (Yang et al., 2016), eggplant (Shibuya et al., 2007, 2008), walnut (Avanzato and Tamponi, 1988) and tomato (Shibuya et al., 2007). However, the molecular basis for the temperature enhancement of regeneration remains poorly characterized. Here, we investigated the effects of temperature upon various aspects of graft healing including callus formation, tissue attachment and vascular formation and revealed a central role for temperature regulating *PIF4* and *YUC2*/*5*/*8*/*9* in leaves to promote vascular formation in grafted stems. Moreover, pharmacological experiments showed that leaf-derived auxin regulated phloem reconnection at the *Arabidopsis* graft junction and xylem bridge formation between the parasite *Phtheirospermum japonicum* and its host *Arabidopsis thaliana* in a temperature-dependent manner. Taken together, our results suggest a conserved temperature signaling mechanism in leaves regulating vascular regeneration and vascular formation in distant tissues.

## RESULTS AND DISCUSSION

### Elevated temperatures enhance vascular formation during grafting

**Fig. 1.**
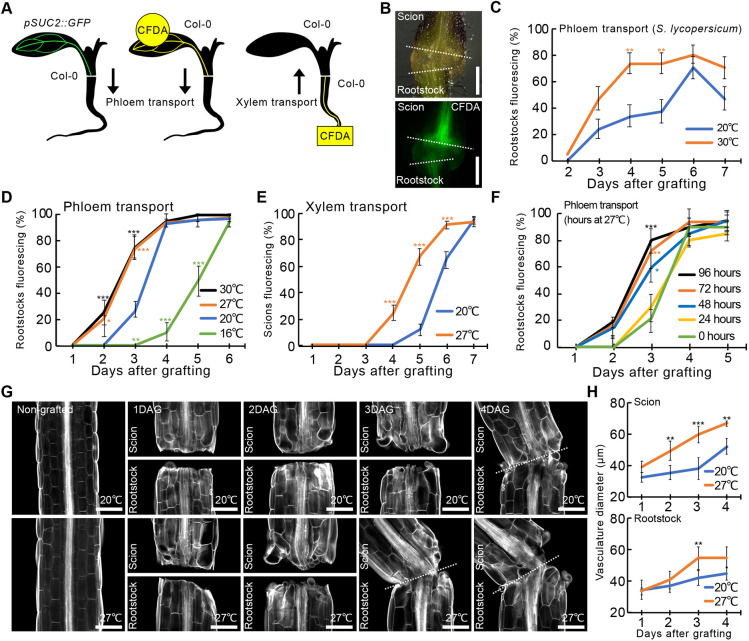
**Elevated temperatures enhance graft formation.** (A) Schematic showing phloem and xylem transport measured by appearance of GFP or CFDA fluorescence in the rootstock (phloem) or CFDA fluorescence in the scion (xylem). (B) Movement of CFDA from scion to rootstock of grafted tomato. Dashed lines indicate cut sites. (C) Proportion of grafted tomato that transported CFDA to rootstocks after recovery at 20°C or 30°C [±standard error of a proportion (s.e.p.); *n*=27 at 1 DAG, *n*=30 at 2-7 DAG per temperature per time point]. (D) Proportion of grafted *pSUC2::GFP Arabidopsis* scions with fluorescing Col-0 rootstocks (±s.e.p.; *n*=20 at 16°C, *n*=60 at 20°C, *n*=20 at 27°C, *n*=20 at 30°C). (E) Proportion of grafted *Arabidopsis* that transported CFDA to scions after recovery at 20°C or 27°C (±s.e.p.; *n*=50 plants per temperature per time point). (F) Proportion of grafted *pSUC2::GFP Arabidopsis* scions with fluorescing Col-0 rootstocks that were recovered at 27°C for the indicated period, then transferred to 20°C recovery (±s.e.p.; *n*=40 plants per temperature). (G) Longitudinal optical sections of grafted *Arabidopsis* recovered at 20°C and 27°C. Plants are stained with Calcofluor White and dashed lines indicate the cut site. (H) Vasculature diameter including pericycle, cambium, xylem and phloem of grafted *Arabidopsis*, 100 µm from the cut surface and recovered at 20°C or 27°C (mean±s.d.; *n*=10 plants per temperature per time point). *P<0.05, **P<0.01, ***P<0.001; Fisher's exact test (C-F) or unpaired two-tailed Student's *t*-test (H) compared with 20°C. Scale bars: 500 μm (B); 100 µm (G).

As elevating temperatures improves commercial grafting success rates (Lagerstedt, 1982), we tested the effects of temperature upon *in vitro* graft formation in tomato (*Solanum lycopersicum*) and *Arabidopsis*. We applied carboxyfluorescein diacetate (CFDA) to monitor vascular connectivity at the graft junction (Melnyk et al., 2015) (Fig. 1A). Tomatoes grown at 25°C and moved to 30°C immediately after grafting showed significantly faster and higher phloem connection rates compared with those recovered at 20°C (Fig. 1B,C). *Arabidopsis* often grafts faster than tomato (Cui et al., 2021; Melnyk et al., 2015; Yin et al., 2012), so we grafted *Arabidopsis pSUC2::GFP* scions to wild-type rootstocks (Fig. 1A) and observed that after 2 days the phloem connection rate was accelerated by higher recovery temperatures (Fig. 1D; Fig. S1), similar to tomato grafting. Expression of a reporter gene associated with cambium formation, *pDOF6::erVENUS* (Smet et al., 2019), was also enhanced by elevated temperatures (Fig. S2A,B). Increasing the recovery temperature from 27°C to 30°C did not further promote *Arabidopsis* graft formation, suggesting that 27°C was close to the maximum thermo-induction effect in *Arabidopsis*. In contrast, reducing the recovery temperature to 16°C delayed phloem reconnection (Fig. 1D). Elevated temperatures also increased xylem reconnection rates (Fig. 1E; Fig. S3) and enhanced the size of the regenerating vascular bundle, particularly in the scion (Fig. 1G,H). We next investigated when and for how long elevated temperatures were required to accelerate graft healing and found that 48 h of warm recovery immediately after grafting was sufficient (Fig. 1F). However, providing warm temperatures before grafting (Fig. S2C) had no significant effect upon vascular connectivity (Fig. S2D), suggesting that thermo-responsiveness occurs early after wounding and plays an important role during graft healing.

### *PIF4* and YUCs are required in the cotyledon for temperature-enhanced vascular formation in the hypocotyl

**Fig. 2.**
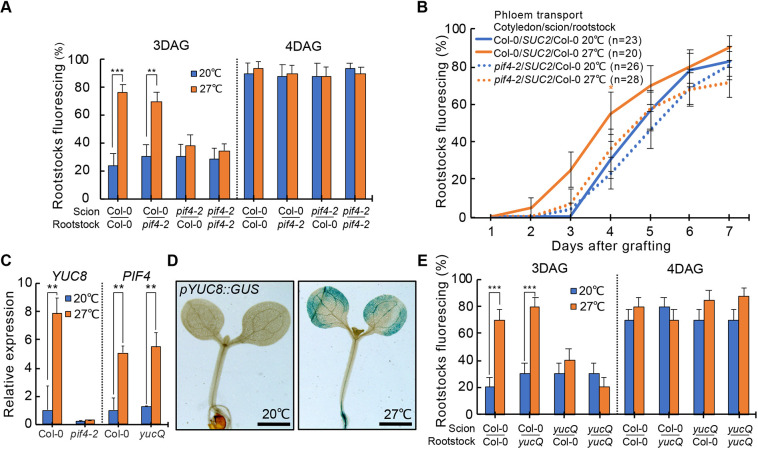
**Temperature-enhanced graft formation requires *PIF4* and YUCs in the cotyledon.** (A) Proportion of grafted *pif4-2* or wild-type *Arabidopsis* that transported CFDA to the rootstock 3-4 DAG and recovered at 20°C or 27°C [±standard error of a proportion (s.e.p.); *n*=30 plants per temperature per time point]. (B) Proportion of three genotype *pif4-2* grafts with fluorescent rootstocks (±s.e.p.; *n*=indicated on plot). (C) Relative expression levels of *YUC8* and *PIF4* in Col-0 or *pif4-2* cotyledons after 48 h treatment of 20°C or 27°C (mean±s.d. of three biological replicates). (D) GUS histochemical staining of 8-day-old *pYUC8::GUS* seedlings incubated at 20°C or 27°C for 48 h. Scale bars: 1 mm. (E) Proportion of grafted *yuc2/5/8/9* or wild-type *Arabidopsis* that transported CFDA to the rootstock, recovered at 20°C or 27°C and measured 3-4 DAG (±s.e.p.; *n*=30 plants per temperature per time point). *P<0.05, **P<0.01, ***P<0.001; Fisher's exact test (A,B,E) or unpaired two-tailed Student's *t*-test (C) compared with 20°C.

To better understand how elevated temperatures promoted graft formation, we tested various mutant genotypes associated with temperature response or hormone signaling (Table S1). Most mutants had no effect on temperature enhancement, but the *pif1 pif3 pif4 pif5* quadruple mutant (*pifQ*) and the *pif4* single mutant were exceptional as they did not respond to temperature enhancement at 3 days after grafting (DAG) but had normal grafting dynamics at later time points (Fig. 2A; Fig. S4A), suggesting they specifically affected temperature enhancement. We tested the spatial requirements of *PIF4* by grafting *pif4* scions to wild-type rootstocks, or vice versa, and observed that *pif4* scions did not respond to the elevated recovery temperature, whereas *pif4* rootstocks responded like wild-type (Fig. 2A). The cotyledons play an important role in thermo-sensing (Bellstaedt et al., 2019), so we generated a graft combination whereby the cotyledon of *pif4* was initially grafted to a *pSUC2::GFP* scion and then, after graft healing, a hypocotyl graft was performed to a wild-type rootstock for recovery at 20°C and 27°C. Plants with *pif4* cotyledons did not respond to elevated temperatures (Fig. 2B), indicating that temperature perception via *PIF4* in the leaves was sufficient to accelerate graft healing in the hypocotyl. The auxin-biosynthesis gene *YUC8* is a direct target of PIF4 (Sun et al., 2012) and we found that *YUC8* transcription levels were upregulated in wild-type plants exposed to elevated temperatures, but downregulated and non-responsive in the *pif4* mutant (Fig. 2C). We also observed that *PIF4* transcript levels were not affected in the *yuc2 yuc5 yuc8 yuc9* quadruple mutant (*yucQ*), consistent with PIF4 acting as an activator of *YUC8*. Staining from *pYUC8::GUS* increased in plants grown at 27°C compared with those grown at 20°C and was observed mainly in the epidermis, vasculature and mesophyll (Fig. 2D), consistent with the previously reported expression pattern of *PIF4* (Kim et al., 2020). We tested the *yucQ* mutant in grafting assays and found that plants lost grafting thermo-responsiveness when YUC genes were mutated in the scion (Fig. 2E), but *yucQ* did not affect grafting at later time points, similar to the *pif4* mutant. The *yucQ* genotype carries *yuc2*, *yuc5*, *yuc8* and *yuc9* mutations yet only *YUC8* was responsive to elevated temperatures (Fig. 2C; Fig. S4B), suggesting that *YUC8* might play a central role for the observed phenotype. We tested whether temperatures affected the expression of vascular-development genes in intact (non-grafted) seedlings but did not detect upregulation (Fig. S4C), suggesting that wounding may be a prerequisite for transcriptional induction. Together, these data indicated a requirement for *PIF4* and YUC genes in the cotyledons for temperature-dependent vascular connectivity in hypocotyl tissues.

### Warm temperatures promote vascular formation by enhancing auxin response

**Fig. 3.**
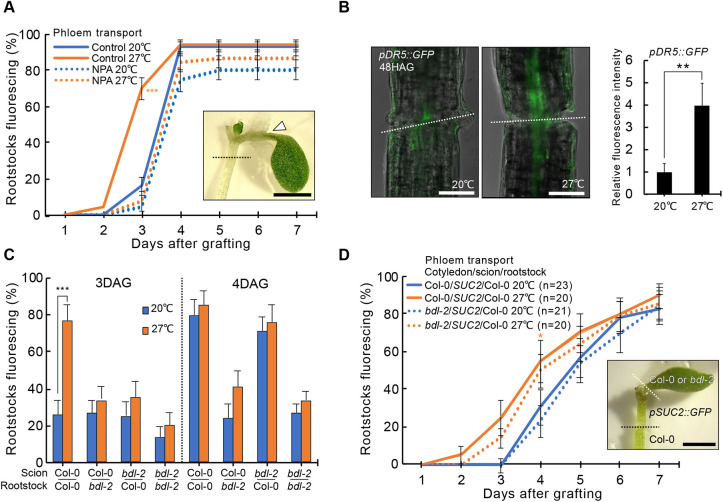
**Temperature promotes graft formation by elevating auxin response.** (A) Proportion of grafted *Arabidopsis pSUC2::GFP* scions with fluorescing Col-0 rootstocks after petiole NPA treatments and recovered at 20°C or 27°C [±standard error of a proportion (s.e.p.); *n*=50 plants per treatment]. NPA plaster (arrowhead) and graft junction (dashed line) indicated. (B) Signal intensity of auxin-responsive *pDR5::GFP* signal at the graft junction 48 h after grafting (HAG) recovered at 20°C or 27°C (mean±s.d. of three experiments, each ≥15 plants per temperature treatment). Dashed lines indicate the graft junction. (C) Proportion of grafted *bdl-2* or wild-type *Arabidopsis* that transported CFDA to the rootstock 3-4 DAG and recovered at 20°C or 27°C (±s.e.p.; *n*=30 plants per temperature per time point). (D) Proportion of three genotype grafts with fluorescent rootstocks (±s.e.p.; *n*=indicated on plot). The white and black dashed lines indicate sites of cotyledon and hypocotyl grafting, respectively. *P<0.05, **P<0.01, ***P<0.001; Fisher's exact test (A,C,D) or unpaired two-tailed Student's *t*-test (B) compared with 20°C. Scale bars: 1 mm (A,D); 100 μm (B).

As auxin is important for graft formation and wound healing (Asahina et al., 2011; Canher et al., 2020; Ikeuchi et al., 2017; Matosevich et al., 2020), we investigated the role of auxin in temperature enhancement of grafting. We applied an auxin transport inhibitor 1-N-naphthylphthalamic acid (NPA) on the petiole to inhibit the transport of auxin from the cotyledon to the hypocotyl, and observed that NPA-treated plants did not respond to temperature enhancement (Fig. 3A), suggesting that cotyledon-derived auxin is essential for this effect. However, graft dynamics of NPA-treated plants at 20°C were similar to controls at 20°C, suggesting that cotyledon-derived auxin was only relevant for graft formation at elevated temperatures. We next asked whether auxin response at the graft junction was increased by elevated temperatures and found a significant fluorescence increase in the auxin-responsive *pDR5::GFP* reporter (Friml et al., 2003; Ulmasov et al., 1997) with warm temperatures (Fig. 3B). Perturbing auxin response in the rootstock with a dominant negative mutant of *BODENLOS* (*BDL*; also known as *IAA12*) (*bdl-2*) (Hayward et al., 2009) blocked graft formation irrespective of whether grafting was performed at 20°C or 27°C (Fig. 3C). However, when *bdl-2* was present only in the scion, plants grafted like controls at 20°C but were inhibited in elevated temperature responses at 27°C (Fig. 3C). As we previously observed that accelerated graft healing in the hypocotyl was due to temperature perception in the leaves, we asked whether blocking auxin response in the leaves would also affect the thermo-responsiveness of grafting dynamics. Blocking auxin response in the cotyledon by grafting the *bdl-2* cotyledon to a *pSUC2::GFP* scion and wild-type rootstock did not affect temperature enhancement (Fig. 3D), suggesting that *bdl-2* did not play a role in the leaves for temperature enhancement of graft formation and, instead, *bdl-2* had its effect at the region of graft junction formation. Thus, the long-distance transport of, and local response to, an auxin-dependent signal was necessary for temperature to accelerate graft healing.

### Temperature-dependent tissue regeneration is widespread

**Fig. 4.**
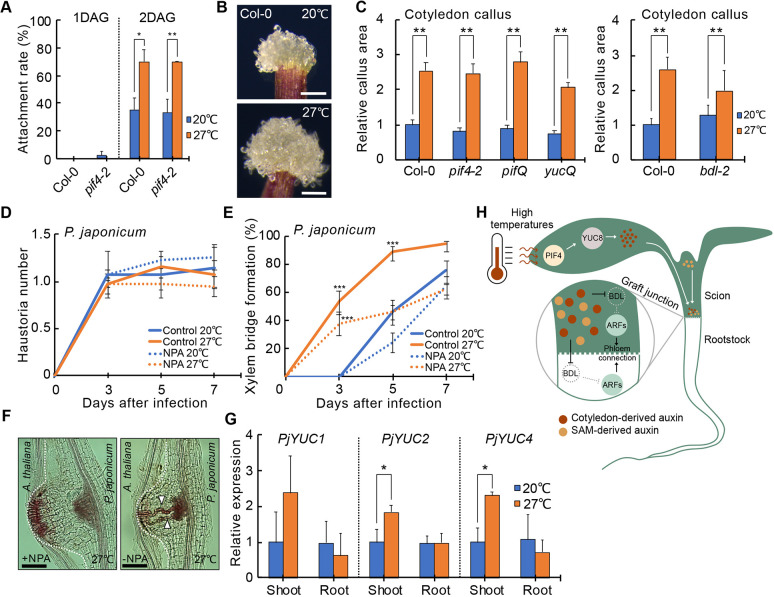
**Temperature-dependent tissue regeneration is widespread.** (A) Proportion of grafts attached 1-2 DAG after recovery at 20°C or 27°C [±standard error of a proportion (s.e.p.); *n*=30 plants per temperature per time point]. (B) Callus formation from cut Col-0 petioles at 20°C or 27°C. (C) Callus size 8 days after wounding from various genotypes relative to Col-0 at 20°C (mean±s.d., *n*=60 cotyledons per genotype and temperature). (D) *P. japonicum* haustoria numbers with control or NPA applications at 20°C or 27°C (mean±s.d. from four experiments, each with 20 infections per treatment). (E) Proportion of *P. japonicum* xylem bridge formation with control or NPA applications at 20°C or 27°C (±s.e.p.; *n*=40 infections per treatment). (F) Representative images of haustoria and xylem bridges formed at 7 DPI at 27°C with and without NPA petiole applications. Dashed lines show the interface between *P. japonicum* and *Arabidopsis.* Xylem bridges are indicated by the arrowheads. (G) Relative expression levels of auxin-related genes in *P. japonicum* at 7 DPI in shoots and roots at 20°C or 27°C (mean±s.d. from three experiments). *P<0.05, **P<0.01, ***P<0.001; Fisher's exact test (A,E) or unpaired two-tailed Student's *t*-test (C,H) compared with 20°C. (H) Proposed model for temperature-dependent vascular regeneration. Elevated temperatures increase PIF4 levels and activate YUC8-mediated auxin production. Auxin moves to the graft junction where it degrades BDL and activates auxin response factors (ARFs) to promote phloem reconnection. SAM, shoot apical meristem. Scale bars: 250 μm (B); 100 μm (F).

Graft formation involves cell adhesion, callus formation and vascular reconnection (Melnyk et al., 2015; Yin et al., 2012). To test the effects of temperature upon tissue adhesion, we picked up plants 1-2 DAG with forceps (Melnyk et al., 2015) and observed that adhesion rates were significantly increased with the elevated temperatures, but this enhancement was not affected in *pif4* mutants (Fig. 4A). To measure callus formation, we used previously described assays (Iwase et al., 2017) and found that elevated temperatures enhanced wound-induced callus formation but this enhancement was not affected in *pif4*, *pifQ*, *yucQ* or *bdl-2* mutants (Fig. 4B,C; Fig. S5A), suggesting that warm temperatures enhanced multiple aspects of wound healing but that phloem enhancement specifically required *PIF4* and *YUC2/5/8/9*.

Parasitic plant infections are conceptually similar to grafting (Kokla and Melnyk, 2018) and their infective structures, haustoria, form xylem connections known as xylem bridges to their hosts to withdraw nutrients. Elevated temperatures can increase haustoria numbers (Rafferty et al., 2019), but we found that elevated temperatures did not affect haustoria number during *A. thaliana* infection by the facultative parasite *P. japonicum* (Fig. S5D). However, we observed that xylem bridge formation was accelerated by elevated temperatures similar to the effect we observed during xylem reconnection at the graft junction (Fig. 1E; Fig. S5E). Warm temperatures also increased the area and length of the haustoria xylem mass adjacent to the parasite root vasculature, the plate xylem (Fig. S5B,C). To investigate a role for leaf-derived auxin, we blocked auxin transport from *P. japonicum* cotyledons using NPA (Fig. S5F). NPA did not affect haustoria number (Fig. 4D), but significantly reduced xylem bridge formation at 27°C but did not affect it at 20°C (Fig. 4E,F). Expression levels of auxin biosynthesis genes *PjYUC2* and *PjYUC4* were upregulated by elevated temperatures in *P. japonicum* shoots but not roots (Fig. 4G; Fig. S5G). Thus, similar to grafting, shoot-derived auxin contributed to vascular formation in basal tissues of *P. japonicum*.

Previous reports have found that elevated temperatures have dramatic effects upon both animal and plant development (Angilletta et al., 2004; Franklin, 2009; Hatfield and Prueger, 2015) and here, we demonstrate that warm temperatures enhanced multiple aspects of wound healing including tissue adhesion, callus formation and vascular regeneration that we could mechanistically separate based on their dependency on *PIF4*. *PIF4* was specifically required in cotyledons to promote vascular regeneration, and this protein is known to activate auxin biosynthesis (Franklin et al., 2011), suggesting that transport of cotyledon-derived auxin was sufficient to enhance vascular formation at the graft junction (Fig. 4H). Enhancing auxin response at the graft junction likely enhanced graft healing through the known roles of auxin in promoting xylem differentiation and activating cambium in part via *DOF6* (Fig. S2) (Baima et al., 1995; Miyashima et al., 2019; Ursache et al., 2014), processes that were likely perturbed in the auxin-resistant *bdl-2* scion (Hayward et al., 2009). However, *bdl-2* rootstocks inhibited grafting regardless of temperature, indicating that the rootstock had a different requirement for auxin response and appeared more sensitive to auxin perturbations. Enhanced temperatures also accelerated haustoria development in *P. japonicum*, and this effect was specific to xylem bridge formation but not haustoria initiation. It was previously shown that auxin production is necessary for haustoria formation and that auxin transport drives xylem bridge formation (Wakatake et al., 2020). We extended the role for auxin and found that shoot-derived auxin acted as a long-distance signal to accelerate xylem bridge formation upon elevated temperatures. Our observations in grafted plants and parasitic plants demonstrate a common mechanism by which temperature sensing in leaves changes vascular development and contributes to modifying the rate of vascular regeneration or vascular formation. Such modulations could provide developmental plasticity in response to environmental changes and confer a fitness advantages to accelerate water and photosynthate transport. High temperatures also enhance regeneration of *Hydra* tentacles (Peebles, 1898), zebrafish fins (Boominathan and Ferreira, 2012) and flatworm testes (Wudarski et al., 2019), suggesting the enhancement of regeneration by elevated temperatures is universally relevant and a useful tool to enhance grafting and wound healing.

## MATERIALS AND METHODS

### Plant materials, growth conditions, and grafting

*A. thaliana* (L.) ecotype Columbia (Col-0) was used throughout this study unless otherwise indicated. Mutant lines used included *pif4-2* (CS66043), *pifQ* (CS66049), *yucQ* (CS69869), *bdl-2* (Hayward et al., 2009). The previously published transgenic lines include *pSUC2::GFP* (Imlau et al., 1999), *pYUC8::GUS* (Müller-Moulé et al., 2016), *pDR5rev::GFPer* (Friml et al., 2003) and *pDOF6::erVENUS* (Smet et al., 2019). For *in vitro* germination, seeds were surface sterilized with 70% (v/v) ethanol for 10 min, followed by 90% (v/v) ethanol for 10 min. The seeds were then sown and germinated on ½MS media (1% plant agar), pH 5.8. After stratification in the dark at 4°C overnight, the seeds were transferred to 20°C short-day growth conditions (8 h of 140 μmol m^−2^ s^−1^). *Arabidopsis* grafting was performed on 7-day-old seedlings and carried out according to previously published protocols (Melnyk, 2017a,b), and recovered at 16°C, 20°C, 27°C or 30°C. For the three-segment cotyledon-hypocotyl grafting, cotyledon grafting was first performed when plants were 4 days old (Bartusch et al., 2020), then after 3 days of recovery at 20°C, the attached plants were used for the hypocotyl grafting. GFP or CFDA signals in the rootstocks were observed daily up to 7 DAG. CFDA signals in the scions were observed daily up to 7 DAG.

MoneyMaker tomato (*S. lycopersicum*) seeds were sterilized in 75% bleach solution for 20 min, then rinsed at least five times with sterile water. The seeds were then sown on ½ MS media (1% agar) and germinated at 25°C under short-day conditions (8 h of 140 μmol m^−2^ s^−1^). Tomato grafting was performed using 7-day-old seedlings. A straight cut was made in the middle of the hypocotyl using a scalpel. Rootstocks and scions were held together within a silicone tube (0.8 mm diameter). The grafted seedlings were transferred on 1% agar media and grown under short-day conditions (8 h of 140 μmol m^−2^ s^−1^), at either 20°C or 30°C. CFDA signals in the rootstocks were observed daily for 7 DAG.

### Phloem and xylem connection assays

Xylem and phloem connections were monitored by the movement of the fluorescent dye CFDA (Thermo Fisher Scientific) across the graft junction. To measure phloem connection, the cotyledon of the *Arabidopsis* grafted plants was wounded with forceps, and then CFDA solution (1 mM) was applied on the surface using a pipette. After 1 h incubation at room temperature, fluorescent signals in the rootstocks were detected. Alternatively, *pSUC2::GFP* (Imlau et al., 1999*)* scions were grafted to wild-type rootstocks, and the GFP signals in the roots were observed daily. For the xylem connection assay, a previously published protocol was modified slightly (Bartusch et al., 2020). In brief, grafted plants with cut root tips were place on a piece of Parafilm, then 1 µl of 1 mM CFDA solution was dropped on the cut site. The signals in the cotyledons were detected after 1 h. For tomato phloem assays, one of the two cotyledons was cut and a drop of CFDA (5 mM CFDA in 1% agar) was applied on the cut site. Seedlings were kept in the dark for at least 2 h. Transversal sections of the hypocotyl (at the shoot-root junction) were made at 2 h and placed on slides to help monitor fluorescence movement. *Arabidopsis* plants and tomato sections were observed under a Leica M205 FA microscope and Leica M205 FCA, with GFP filter to detect CFDA fluorescence in the phloem or xylem. All of the CFDA assays were performed at room temperature. Ungrafted plants were used as controls.

### Parasitic plant infection assays

*P. japonicum* seeds were surface sterilized by washing with 70% ethanol for 20 min, followed by 95% ethanol for 5 min, and sown on ½ MS with 1% sucrose and 0.8% agar. After stratification at 4°C in darkness overnight, the plates were moved to a growth cabinet at 20°C in short-day conditions (8 h of 140 μmol m^−2^ s^−1^). Four-day-old *P. japonicum* seedlings were transferred to 0.8% water agar for starvation before infection. For the infection, the root of a 5-day-old *Arabidopsis* seedling was aligned to each *P. japonicum* root, and the infection setup was incubated at 20°C or 27°C in short-day conditions (8 h of 140 μmol m^−2^ s^−1^). At 24 h post infection, swellings on *P. japonicum* root corresponding to early haustoria were marked as day-1 haustoria. The number of haustoria and presence of a xylem bridge were quantified using a Zeiss Axioscope A1 microscope at 3, 5 and 7 days post infection (DPI). Plate xylem area and length were measured on 7 DPI haustoria stained with Safranin-O using a previously published protocol (Spallek et al., 2017).

### Histological staining and confocal imaging

Histological staining of GUS was analyzed in *pYUC8::GUS* transgenic seedlings, which were germinated and grown at 20°C for 6 days, then transferred to 27°C, or remained at 20°C, for 48 h. For the staining, seedlings were incubated for 12 h at 37°C with the substrate solution (1 mM 5-bromo-4-chloro-3-indolyl-β-D-glucuronide, pH 7.0, 100 mM sodium phosphate buffer, 10 mM Na_2_EDTA, 0.5 mM potassium ferricyanide, 0.5 mM potassium ferrocyanide and 0.1% Triton X-100). Stained seedlings were washed with 70% ethanol overnight to remove chlorophyll, and were then photographed using a Leica M205 FA microscope. For confocal microscopy, all images were taken on a Zeiss LSM-780 laser scanning confocal microscope. Graft junction morphology was observed with Calcofluor White staining protocol (Ursache et al., 2018), with 405 nm excitation, 2% laser power, 410-529 nm detection and 210 PMT. Vascular diameter quantifications included cambium, xylem, phloem and pericycle tissues and measured the distance between the pericycle layers encompassing the vascular bundle, 100 μm above the cut site. Samples with GFP and Venus were excited with 488 nm excitation, 10% laser power, 500-524 nm detection and 280 PMT. The images were processed and analyzed using FIJI software (version 2.1.0/1.53c).

### NPA treatment assay

The application of the auxin inhibitor NPA plasters on *Arabidopsis* was adapted from a previous study (Bellstaedt et al., 2019). Plants were grown in short-day conditions at 30 μmol m^−2^ s^−1^ to induce longer petioles before grafting, for a more efficient application of NPA plasters. Thin strips of cellulose tissue were soaked in a lukewarm agar solution (1%) with or without 100 µM NPA (Duchefa) and were carefully positioned across petioles using fine forceps after grafting. For the application of NPA on *P. japonicum*, the NPA plasters were placed on the cotyledons just before the infection assay.

### Plant attachment and callus formation assays

For the attachment assays, grafted *Arabidopsis* recovered at 20°C to 27°C were picked up with forceps at the root/hypocotyl junction and scions scored whether they remained attached or fell apart. The petiole callus formation assays was adapted from previously published protocols (Iwase et al., 2017). The explants were incubated at 20°C to 27°C and the area of callus was quantified by ImageJ (version 2.1.0/1.53c). Callus induction was quantified as a percentage of explants with more than one callus cell developing from wound sites.

### Gene expression analyses

For *Arabidopsis*, total RNA was extracted from whole seedlings or cotyledons using ROTI Prep RNA MINI (Roth), then subsequently treated with DNase I (New England Biolabs) to eliminate DNA contamination. The cDNA was synthesized with Maxima First Strand cDNA Synthesis Kit for RT-qPCR (Thermo Fisher Scientific). The transcript levels were measured by quantitative real-time PCR (qPCR) using SYBR-Green master mix (Applied 512 Biosystems) with specific primers (Table S2). The data were normalized against temperature-stable housekeeping gene *PP2A* (Hong et al., 2010). The temperature-stable housekeeping gene *MONENSIN SENSITIVITY1* (*MON1*) transcript levels remained unchanged at 20°C and 27°C in Col-0, *pif4-2* and *yucQ* (Fig. S4D). The relative expression was calculated using the Pfaffl method (Pfaffl, 2001). All reactions were carried out with three biological replicates, each with three technical replicates. For *P. japonicum*, 7 DPI plants were separated from host *Arabidopsis*, and the shoot and root samples were collected. Total RNA extraction, cDNA synthesis and qPCR were performed using the mentioned protocol with *P. japonicum*-specific primers (Table S2). The data were normalized against *PjPP2A*, the homolog of *AtPP2A*.

### Statistics

For pairwise comparisons of frequencies, Fisher's exact test was used with the indicated sample sizes. For pairwise comparisons of continuous data, unpaired two-tailed Student's *t*-test was performed.

## Supplementary Material

Supplementary information

Reviewer comments
