# Supplementary material for: High temperature perception in leaves promotes vascular regeneration and graft formation in distant tissues
Source: Development. 2022 Feb 28;149(5):dev200079. doi: 10.1242/dev.200079 (PMC8959136; doi:10.1242/dev.200079)
Supplement: Supplementary information [file develop-149-200079-s1.pdf]

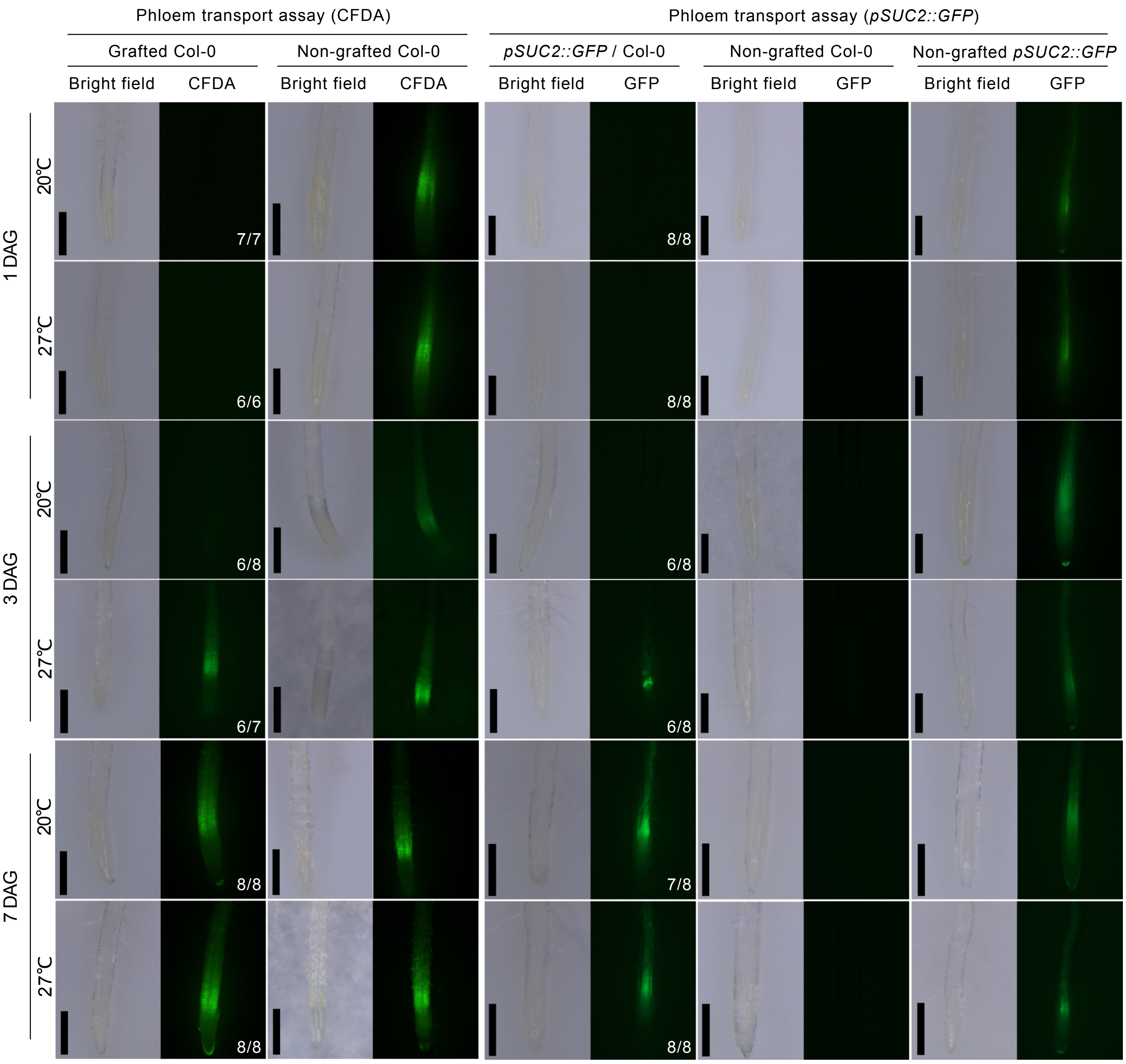

Fig. S1. Phloem connectivity assays. Detection of fluorescence at the root tip of plants recovered at 20℃ or 27℃. The number at the bottom right represents the proportion of the shown individuals at 1, 3, and 7 DAG. Scale bars=200 μm.

Development • Supplementary information

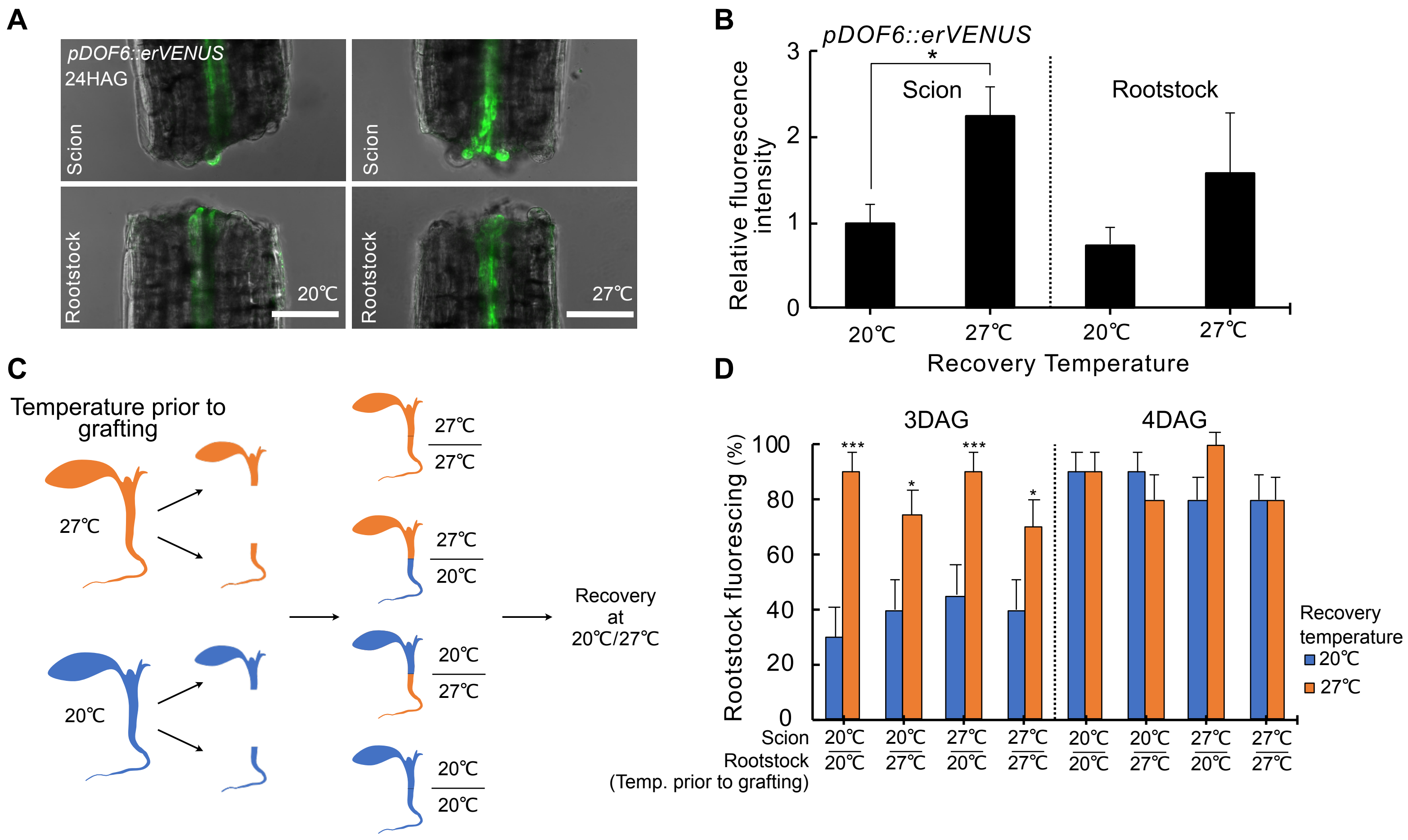

Fig. S2. Elevated temperatures are required during graft recovery. (A) Confocal images of *pDOF6::erVENUS* scions and rootstocks of plants recovered at 20℃ or 27℃ at 24 hours after grafting (HAG). Plants were grafted but not firmly attached during imaging at 24 hours so scions and rootstocks are shown separately. Scale bars=100 µm. (B) Signal intensity of *pDOF6::erVENUS* signal at the graft junction recovered at 20°C or 27°C (mean ±s.d. of three experiments, each ≥15 plants per temperature treatment). **P*< 0.01; unpaired two-tailed Student's *t*-test. (C) Col-0 seedlings cultivated for 7 days at 20℃ or 27℃ were cut and grafted with various temperature combinations. The grafted plants were subsequently transferred to recover at 20℃ or 27℃. (D) Proportion of grafted wild-type *Arabidopsis* that transported CFDA to the rootstock 3-4 DAG and recovered at 20°C or 27°C. The combination of temperature treatment prior to grafting is indicated (±s.e.p.; n=20 plants per temperature per time point). **P*<0.05; ***P*<0.01; ****P*<0.001; Fisher's exact test.

Development • Supplementary information

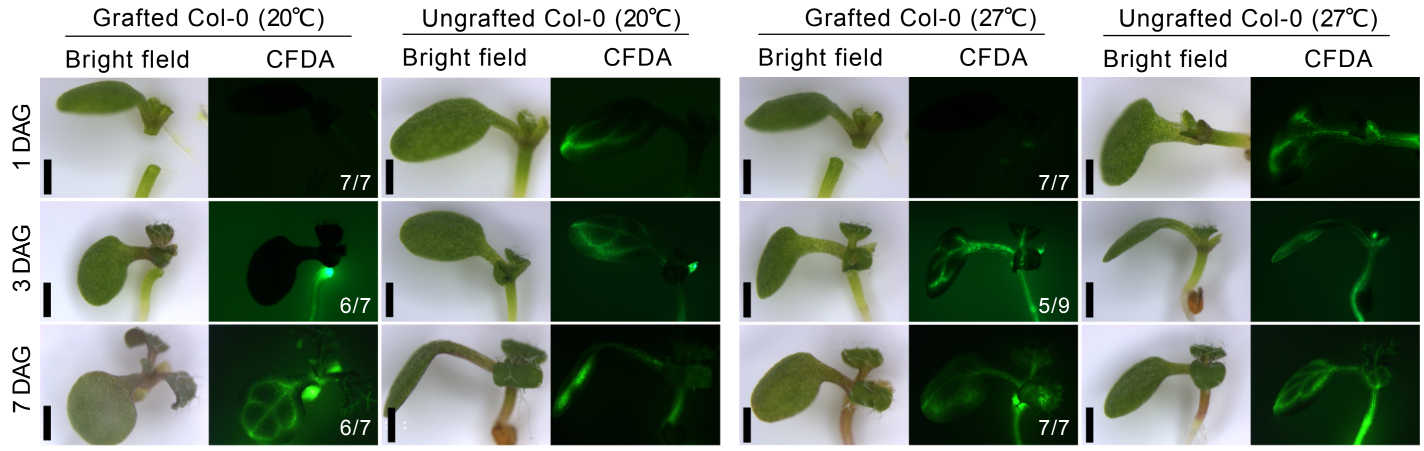

Fig. S3. Xylem connectivity assays. Detection of fluorescence at the shoot of plants recovered at 20℃ or 27℃. The number at the bottom right represents the proportion of the shown individuals at 1, 3, and 7 DAG. Scale bars=500 μm.

Development • Supplementary information

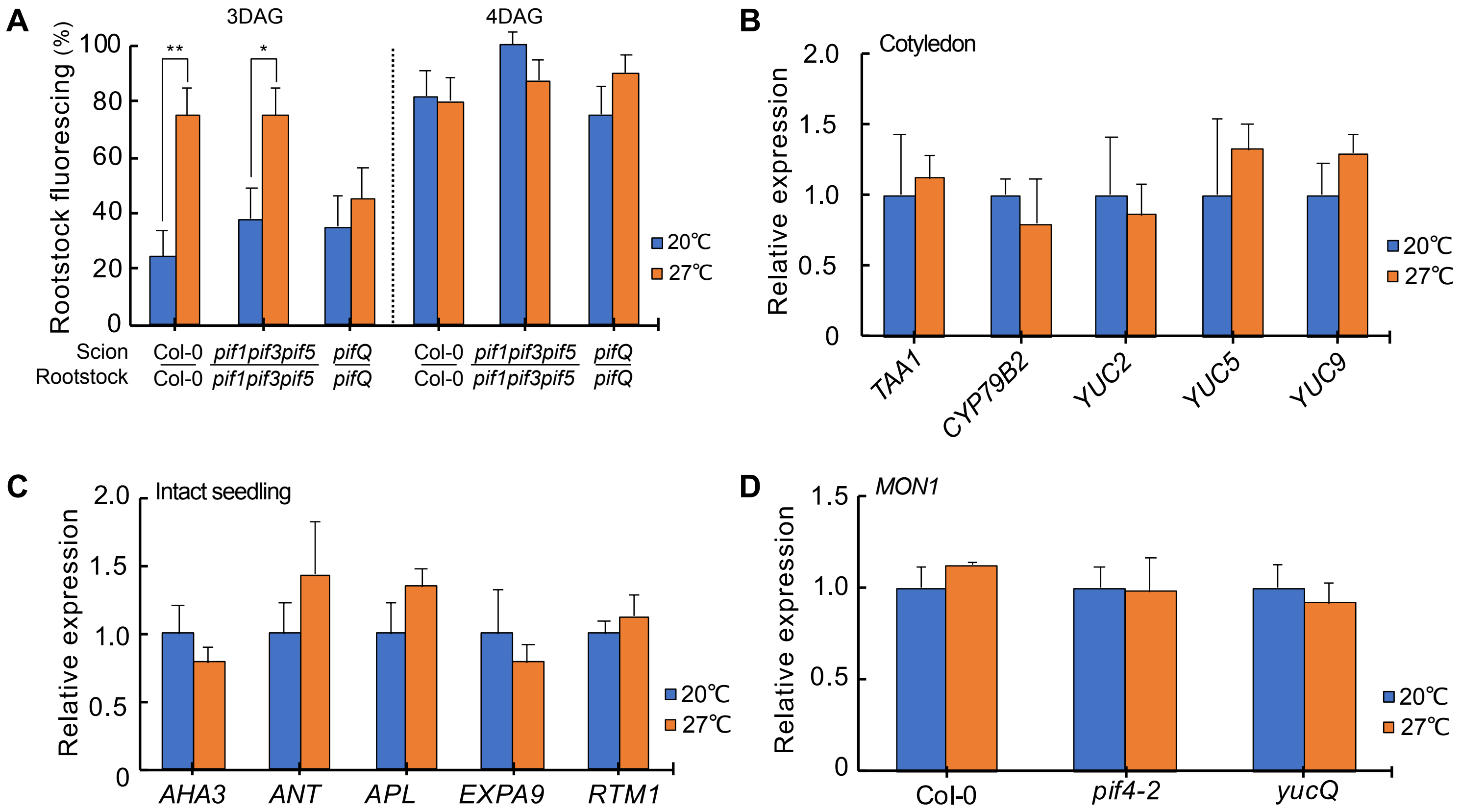

Fig. S4. Grafting of *pif* mutants and expression analysis of selected genes.

(A) Proportion of grafted wild-type, *pif1pif3pif5*, or *pif1pif3pif4pif5* (*pifQ*) *Arabidopsis* that transported CFDA to the rootstock 3-4 DAG and recovered at 20°C or 27°C (±s.e.p.; n=20 plants per temperature per time point). **P*<0.05; ***P*<0.01; ****P*<0.001; Fisher's exact test. (B) Relative expression levels of selected auxin biosynthesis genes in the cotyledons of 7 day-old Col-0 incubated at 20℃ and 27℃ for 48 hours. (C) Relative expression levels of selected vascular genes in 7 day-old Col-0 seedlings incubated at 20℃ and 27℃ for 48 hours. (D) Relative expression levels of temperature-stable housekeeping gene *MON1* in 7 day-old seedlings incubated at 20℃ and 27℃ for 48 hours. (B-D) The expression levels of each gene is normalized to expression at 20℃ (mean±s.d. of three biological replicates). Unpaired two-tailed Student's *t*-test was performed, and the data show no significant difference compared to 20℃ (*P*<0.01).

Development • Supplementary information

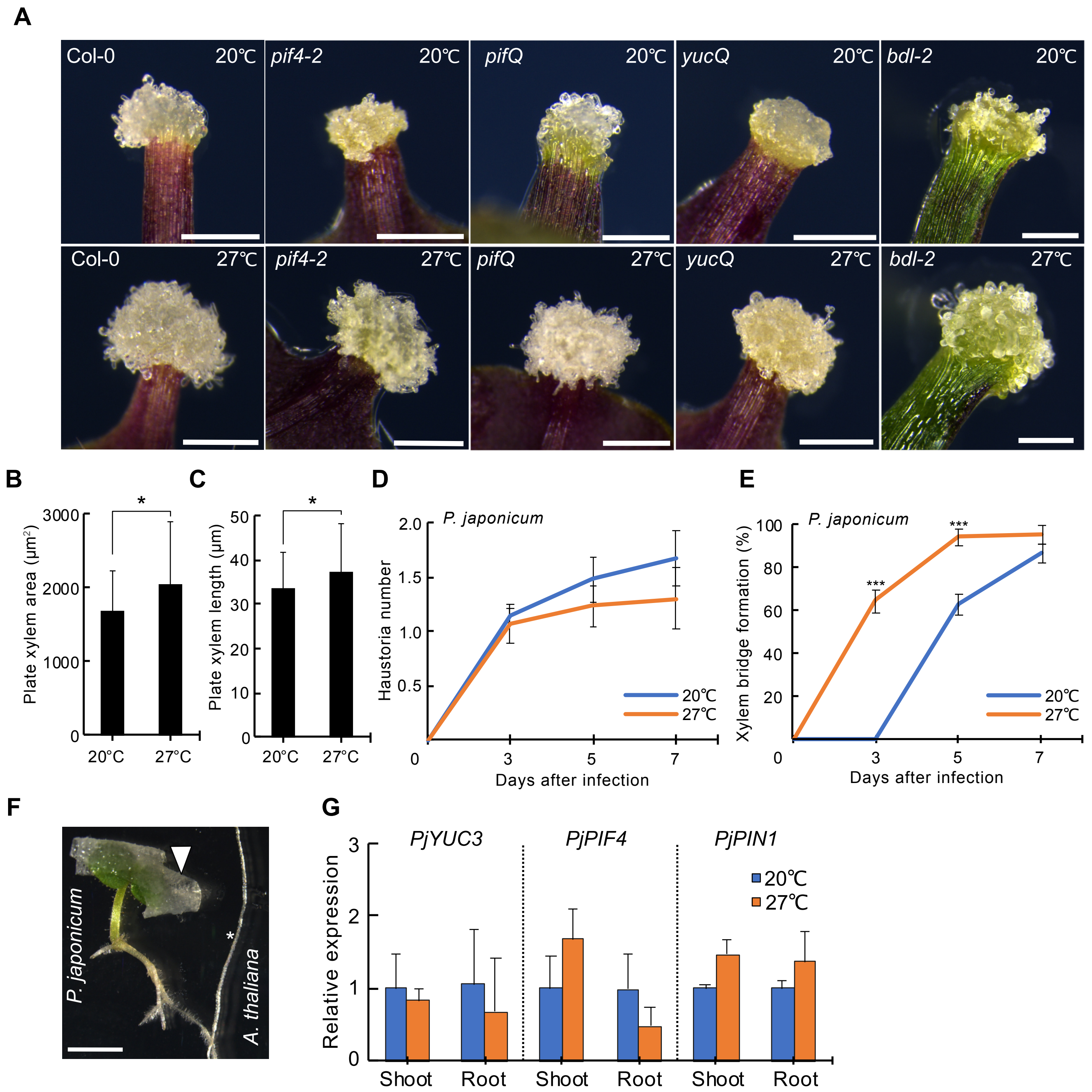

Fig. S5. Elevated temperatures promote several aspects of tissue regeneration. (A) Callus formation from cut petioles at 20°C or 27°C. Scale bars=500 μm. The images are representative of the average of 60 leaves from each genotype and temperature treatment. Col-0 taken from Fig.4B. (B-C) Measurement of plate xylem area (B) and length (C) in the haustoria of the parasitic plant *P.japonicum* infecting the host plant *Arabidopsis* at 7 DPI at 20℃ or 27℃. The value indicates the mean (±s.d.) of 74 haustoria per temperature treatment. **P*<0.01; unpaired two-tailed Student's *t*-test. (D) Haustoria number of *P.japonicum* infecting *Arabidopsis* at 20°C and 27°C (mean±s.d. from four experiments, n= 20 infections per treatment). (E) Proportion of *P.japonicum* xylem bridge formation at 20°C or 27°C (±s.e.p.; n= 40 infections per treatment). (F) Representative images of cotyledon NPA application on *P.japonicum*. The white arrow head indicates the site of the NPA plaster. The asterisk indicates the root of the *Arabidopsis* host. (G) Relative expression levels of auxin-related genes in the parasitic plants at 7 DPI in the shoots and roots at 20℃ or 27℃(mean±s.d. from three experiments). Unpaired two-tailed Student's *t*-test was performed, and the data show no significant difference compared to 20℃ (*P*<0.01).

Development • Supplementary information

Table S1. Genotypes tested for phloem connection. 7-day-old seedlings were self-grafted (unless indicated) and recovered at 20°C or 27°C. Phloem reconnection measurement was done by CFDA assay at 3 and 4 DAG. The value indicates percentage of rootstocks fluorescing of at least 20 plants. Asterisks indicate the heterograft with Col-0 rootstocks.

		Rootstock fluorescing (%)				
		3DAG		4DAG		
Genotype	Background	20℃	27℃	20℃	27℃	Genotype reference
Col-0		30	80	80	90	
Ler		10	30	55	65	
alf4-2 /Col-0*	Col-0	20	60	80	90	Bagchi et al. 2018. The EMBO journal. 37(2), 255–268.
arf6-2	Col-0	50	70	60	85	Nagpal et al. 2005. Development. 132, 4107-4118.
arf7-1 arf19-1	Col-0	30	90	85	95	Okushima et al. (2005). Plant Cell. 17, 444-463.
arf8-3	Col-0	30	75	70	75	Nagpal et al. 2005. Development. 132, 4107-4118.
arp6-1	Col-0	30	80	65	90	Deal et al. 2005. Plant cell. 17(10), 2633–2646
axr1-12 /Col-0*	Col-0	10	80	40	80	Lincoln et al. 1990. Plant Cell. 2, 1071-1080.
axr4-2	Col-0	20	90	80	90	Hobbie et al. 1995. The Plant Journal. 7, 211-220.
hy5-51	Col-0	12	54	50	80	Ruckle et al. 2007. Plant Cell. 19(12), 3944–3960.
iaa3 (shy2-2)	Ler	30	40	65	70	Reed et al. 1998. Genetics. 148, 1295-1310.
iaa7 (axr2-1)	Col-0	0	70	50	90	Wilson et al. 1990. Molecular & General Genetics. 222, 377-383.
iaa12 (bdl-2)	Col-0	35	45	75	90	Hayward et al. 2009. Plant Physiology. 151, 400-412.
iaa14 (slr1)	Col-0	0	90	60	80	Fukaki et al. 2002. The Plant Journal. 29, 153-168.
iaa19 (msg2-1)	Col-0	30	70	70	80	Tatematsu et al. 2004. Plant Cell. 16, 379-393.
pif4-2	Col-0	30	35	98	90	Leivar et al. 2008. Plant Cell. 20(2), 337-52.
pif1-1 pif3-7 pif5-3	Col-0	37	75	95	90	Leivar et al. 2008. Current Biology.18(23),1815-1823.
pifQ (pif1-1 pif3-7 pif4-2 pif5-3)	Col-0	35	45	75	90	Leivar et al. 2008. Current Biology.18(23),1815-1823.
35S::PIF4-HA	Col-0	0	0	0	0	Lorrain et al. 2008. Plant Journal. 53(2), 312-323.
tir1-1 afb2-3	Col-0	0	25	30	75	Parry et al. 2009. PNAS. 106, 22540- 22545.
wei8 tar1-1	Col-0	5	50	30	75	Stepanova et al. 2008. Cell. 133(1):177-191.
wei8-4	Col-0	0	50	45	90	Stepanova et al. 2008. Cell. 133(1):177-191.
yucQ (yuc2 yuc5 yuc8 yuc9)	Col-0	30	20	70	88	Müller-Moulé et al. 2016. PeerJ. 4, e2574.
yuc1D	Col-0	0	20	15	60	Zhao et al. 2001. Science. 291:306–9.
yuc1D /Col-0*	Col-0	20	20	30	30	Zhao et al. 2001. Science. 291:306–9.
35S::AtPHYB	Col-0	0	0	5	15	Su and Lagarias. 2007. Plant Cell. 19(7):2124-39.
phyB-9	Col-0	15	25	55	90	Reed et al. 1993. Plant Cell. 5(2), 147–157
pgp1 pgp19	Col-0	5	20	25	40	Geisler et al. 2003. Molecular Biology Cell. 14:4238–4249.
pgp4-1	Col-0	5	35	15	65	Terasaka et al. 2005. Plant Cell. 17(11), 2922-2939.
pin1-1	Col-0	5	35	50	70	Okada et al 1991. Plant Cell. 3:677.
pin3-3 pin4-3 pin7-1	Col-0	0	15	0	15	Bennett et al. 2016. PLoS Biology. 14, e1002446.
aux1 lax1 lax2 lax3	Col-0	0	5	5	25	Bainbridge et al. 2008. Genes Development. 22(6), 810-823.
35S::bzr1-1D	Col-0	40	85	95	95	Oh et al 2014. Nature Communication. 5:4140.
bes1D	Col-0	0	5	20	0	Yin et al. 2002. Cell. 109(2):181-191.

Table S2. Primer sequences used for qPCR.

Gene	Direction	Sequence (5'-3')	Gene ID	GenBank accession
AHA3	Forward	GCTGGTATGGATGTTCTGTGC	AT5G57350	
AHA3	Reverse	GGTTCGCTTATCAACTGGATT	AT5G57350	
ANT	Forward	GATGTAGCAGCAATTAAGTTCCG	AT4G37750	
ANT	Reverse	GAGCGGTTTGGTCTTCAGTATT	AT4G37750	
APL	Forward	ACCAAGTCCTCGACCATCACA	AT1G79430	
APL	Reverse	CTCCGACAAAGAATCCAAATCC	AT1G79430	
TAA1	Forward	ACGCAAACGCAGGGTAAGA	AT1G70560	
TAA1	Reverse	AGATGATGAAGAATCGGTGGG	AT1G70560	
CYP79B2	Forward	CATCGACGAGAGGATCAAGATGTGG	AT4G39950	
CYP79B2	Reverse	TTGGGATGTCGGATTCTTGAACGAG	AT4G39950	
EXPA9	Forward	TGATCTCGCTATGCCTATGTTT	AT5G02260	
EXPA9	Reverse	TGACCAGCACCAAGTTGAAGTA	AT5G02260	
PIF4	Forward	GCCAAAACCCGGTACAAAACCA	AT2G43010	
PIF4	Reverse	CGCCGGTCAACTAAATCTCAACATC	AT2G43010	
RTM1	Forward	ATTGTAAACTGGGACGAAGGAT	AT1G05760	
RTM1	Reverse	CGTACTCGCTAGTGTTGGTATTG	AT1G05760	
YUC2	Forward	ATTGTCTCATCCTTCTTCCCTC	AT4G13260	
YUC2	Reverse	TTCGTCCAATACCTTGAGTCTTAC	AT4G13260	
YUC5	Forward	CATTAGCATTGTGATTTGCGAGAT	AT5G43890	
YUC5	Reverse	GGAGTTTGAAGGCGAGGTGA	AT5G43890	
YUC8	Forward	AAACGCTCAAGGGGTTCTCTTCG	AT4G28720	
YUC8	Reverse	CACGCACAACACCCTTTGATTCG	AT4G28720	
YUC9	Forward	AAGGAGTCCCATTCGTTGTG	AT1G04180	
YUC9	Reverse	CGTTGGGTATTCAGGGTAGTG	AT1G04180	
MON1	Forward	CAGACAAGGCGATGGCGATA	AT2G28390	
MON1	Reverse	GCTTTCTCTCAAGGGTTTCTGGGT	AT2G28390	
PjPIF4	Forward	GCGTCGTAAAACCCGACAAC	Pjv1_00017700	BMAC01000436
PjPIF4	Reverse	CGAAGCCCAACTCTTGTCCA	Pjv1_00017700	BMAC01000436
PjYUC1	Forward	CTACGCCAGCCACTTCTCAA	Pjv1_00018474	BMAC01000416
PjYUC1	Reverse	GATTTCCGGTATCACGGGCT	Pjv1_00018474	BMAC01000416
PjYUC2	Forward	AAATTCCGGCATGGAGGTGT	Pjv1_00010538	MN064668
PjYUC2	Reverse	CACGAGCCATAGTGGTAGCC	Pjv1_00010538	MN064668
PjYUC3	Forward	TTTGGGTAGTGGACCGGGTA	Pjv1_00016159	MN064669
PjYUC3	Reverse	TCAGCCCATGCTCTTCTGTG	Pjv1_00016159	MN064669
PjYUC4	Forward	GCCTTAAAGAGCATGGCGTC	Pjv1_00003857	MN064670
PjYUC4	Reverse	ATTGCTTGGGGAGATGGAGC	Pjv1_00003857	MN064670
PjPIN1	Forward	AAGTGCAAGTGCGAGCTTTG	Pjv1_00008680	BMAC01000122
PjPIN1	Reverse	TTTGGGGTACAGTGGTGGTG	Pjv1_00008680	BMAC01000122
PjPP2A	Forward	GGGGTCTTTCACCCTCACTC	Pjv1_00012240	BMAC01000248
PjPP2A	Reverse	CATGCGGAACCTCCTGTGTA	Pjv1_00012240	BMAC01000248

Development • Supplementary information
