# Supplementary material for: High temperature perception in leaves promotes vascular regeneration and graft formation in distant tissues
Source: Development. 2022 Feb 28;149(5):dev200079. doi: 10.1242/dev.200079 (PMC8959136; doi:10.1242/dev.200079)
Supplement: Reviewer comments [file develop-149-200079_review_history.pdf]

High temperature perception in leaves promotes vascular regeneration and graft formation in distant tissues

Phanu T. Serivichyaswat, Kai Bartusch, Martina Leso, Constance Musseau, Akira Iwase, Yu Chen, Keiko Sugimoto, Marcel Quint and Charles W. Melnyk

DOI: 10.1242/dev.200079

Editor: Yka Helariutta

Review timeline

Original submission:	6 August 2021
Editorial decision:	20 September 2021
First revision received:	6 December 2021
Accepted:	21 January 2022

Original submission

First decision letter

MS ID#: DEVELOP/2021/200079

MS TITLE: High temperature perception in leaves promotes vascular regeneration in distant tissues

AUTHORS: Phanu T Serivichyaswat, Kai Bartusch, Martina Leso, Constance Musseau, Akira Iwase, Yu Chen, Keiko Sugimoto, Marcel Quint, and Charles W. Melnyk

I have now received all the referees' reports on the above manuscript, and have reached a decision. The referees' comments are appended below, or you can access them online: please go to BenchPress and click on the 'Manuscripts with Decisions' queue in the Author Area.

As you will see, the referees express considerable interest in your work, but have some significant criticisms and recommend a substantial revision of your manuscript before we can consider publication. If you are able to revise the manuscript along the lines suggested, which may involve further experiments, I will be happy receive a revised version of the manuscript. Your revised paper will be re-reviewed by one or more of the original referees, and acceptance of your manuscript will depend on your addressing satisfactorily the reviewers' major concerns. Please also note that Development will normally permit only one round of major revision.

We are aware that you may be experiencing disruption to the normal running of your lab that make experimental revisions challenging. If it would be helpful, we encourage you to contact us to discuss your revision in greater detail. Please send us a point-by-point response indicating where you are able to address concerns raised (either experimentally or by changes to the text) and where you will not be able to do so within the normal timeframe of a revision. We will then provide further guidance. Please also note that we are happy to extend revision timeframes as necessary.

Please attend to all of the reviewers' comments and ensure that you clearly highlight all changes made in the revised manuscript. Please avoid using 'Tracked changes' in Word files as these are lost in PDF conversion. I should be grateful if you would also provide a point-by-point response detailing how you have dealt with the points raised by the reviewers in the 'Response to Reviewers' box. If you do not agree with any of their criticisms or suggestions please explain clearly why this is so.

© 2022. Published by The Company of Biologists under the terms of the Creative Commons Attribution License (https://creativecommons.org/licenses/by/4.0/).

Reviewer 1

Advance summary and potential significance to field

The manuscript “High temperature perception in leaves promotes vascular regeneration in distant tissues” subjects the temperature-induced effect on vascular development in Arabidopsis and tomato seedlings after grafting.
The aspect of still elusive machinery of vasculature healing after wounding or grafting is very intriguing. The role for many external and internal factors, including phytohormones and signaling pathways in response to incision remains far unknown. Thus, I have read the manuscript with interest. I find this a well written manuscript, with well executed and presented results. The manuscript is relevance for better understanding how plants undertake the process of grafting in aspect of their ability to cellular response and vascular tissue regeneration under elevated temperature. The Authors research is valuable and bring new insight into this complicated process. I appreciate the work the Authors put into this paper.

Comments for the author

However, I have some comments and questions to the Authors:
1. Experiments were performed with tomato and Arabidopsis seedlings. The Authors observed that increasing the recovery temperature from 27ºC to 30ºC did not promote Arabidopsis graft formation, suggesting that 27ºC is close to the maximum thermo-induction effect (lines 123-125, Results), whereas in experiments with tomatoes observed that grafted seedlings recovered at 30ºC showed significantly faster and higher phloem connection rates (lines 117-118, results). In turn, in Arabidopsis pSUC::GFP and DOF6::Venus lines cambium formation and phloem connection rate was similar to tomato grafting. How could it be understand? Could I ask about short comment to your observations? I think it should be clarified and precisely described in Results.
2. Cambium, the lateral meristem responsible for the vascular development plays important role during the vasculature healing after grafting or wounding. Is it possible to include some microscope images showing the cambium response (i.e. higher/lower cambial cell divisions) on the elevated temperature induction? It would enrich very much the observed changes during vascular regeneration in grafted seedlings and results presented in the manuscript. I will be very satisfied you supplement your results with cambium reaction.
3. Fig. 2D shows GUS staining of pYUCCA8::GUS Arabidopsis line, grown at 20ºC and 27ºC for 48h. GUS reaction has not been observed on the image with the seedling grown at 20ºC. I expect poor signal, but not lack of the YUC8 expression. Could the Authors comment these results?
4. Slight deficiency of the manuscript is unclear experimental establishing in some point of the described methodology. Following questions:
4.1. How old seedlings were used for the analyses? I have found in the Material and methods that: ‘for three-segment cotyledon-hypocotyl grafting, cotyledon grafting was first performed when plants were four days old, then after three days of recovery at 20ºC, the attached plants were used for the hypocotyl grafting’ (lines 282-285, M&M); ‘tomato grafting was performed using seven-day-old seedlings’ (line 290, M&M); GUS reaction was analyzed in 8-day-old seedlings and for the CFDA treatment I could not found how old seedlings were used. It should be clarified in the M&M chapter.
4.2. Number of analyzed plants per each of the performed experiment is confused, i.e. n=40-80 plants per each of temperature treatment (Fig.1D) or n=30-45 plants (Fig.2A, E; Fig.3C) etc. In my opinion this is not precise and make unclear how many plants were finally analyzed per each of the experiment. I suggest to add ‘respectively’ in such record or simply assign one number of plants used/analyzed in each of experiment. Other suggestion – it should be clarified in M&M chapter.
5. Maybe it would be worth to discuss the temperature-induced effect on vascular tissue regeneration in grafting seedlings with other works on the vasculature healing after wounding (i.e. Sauer et al. 2006, Genes Dev. 20: 2902-2911; Balla et al. 2011, Plant J. 65: 571-577; Hajny et al. 2020, Science)?

Minor points
1. Line 62, 172 – dots in the middle of the sentences.
2. Should be used the first letter of the name during citation? – lines 62, 97 in the text.
3. Fig. 3A – lack of the ‘days after grafting’ in the graph axis description.

© 2022. Published by The Company of Biologists under the terms of the Creative Commons Attribution License (https://creativecommons.org/licenses/by/4.0/).

Reviewer 2

Advance summary and potential significance to field

This manuscript shows a link between temperature perception and some aspect of vasculature regeneration.
The cases studied are focused on tissue grafting, host-pathogen connection and callus formation: although quite specific examples of tissue self-organization, they offer a relevant set of independent developmental scenarios where temperature perception is shown to play a role.
The presented data is sound and, besides a few possible improvements suggested below, will be significant for developmental biologists interested in tissue regeneration, self-organization, auxin signalling and temperature sensing.
I have no doubt that this manuscript is appropriate for the Development readership.

Comments for the author

From the general to the specific:
1. The grafting experiments heavily depend on the methodological approach of inferring vascular continuity from fluorescent dye (CFDA) or protein (GFP) diffusion. Although this analysis of functional vasculature regeneration “improved” at high temperature is certainly valid and important, the argument would be significantly strengthen by a parallel morphological analysis of the grafting zone. The authors should consider including time-lapse, high-resolution images of vasculature regeneration at low/high temperature, to correlate with the functional data already presented.
2. Related to the point above, is it possible that high temperature is simply increasing the CFDA/GFP diffusion rate through the tissue, rather than tissue regeneration? The authors should include controls at low/high temperature with not-grafted (i.e. intact) seedlings, at least for the CFDA experiments and perhaps with transgenic line expressing GFP only in the leaf phloem?
3. Regarding the expression analysis (e.g. Fig 2C or Fig S2B,C), is it possible that high temperature in these conditions increases a wide range of non-specific transcriptional activity? The authors should present data about the relative expression (high/low temperature) of some housekeeping genes, as negative controls.
4. The error in the estimate of a proportion from a sample can be quantified with a “standard error of the proportion”, or s.e.p.). Although the statistical test when comparing proportions used by the authors seems appropriate and their conclusions correct, they should add error bars with s.e.p. in all their graphs reporting estimated proportions.

Reviewer 3

Advance summary and potential significance to field

In this manuscript by Serivichyaswat et al., the authors connect the phenomenon of heat-enhanced graft formation with molecular regulators for auxin biosynthesis response, and temperature perception. The authors use refined cotyledon grafting to demonstrate that auxin-dependent temperature perception and response occurs in distant leaf tissues, rather than locally at the graft interface. Furthermore they extend their model beyond grafting, by demonstrating that plant parasite haustorium formation is also enhanced by elevated temperatures. Overall, this paper is relevant to work on regeneration biology and agricultural research on grafting, and thus of broad interest to the research community. The experimental methods are generally robust, although I list some suggestions that I think would improve the study below, and I’m particularly impressed by the cotyledon grafting.

Comments for the author

Comments:
Successful graft formation in this study is based on resumed physiological xylem and phloem transport. I would like to see this paired with some detailed anatomical verification of vascular connections within the graft junction.

© 2022. Published by The Company of Biologists under the terms of the Creative Commons Attribution License (https://creativecommons.org/licenses/by/4.0/).

In the discussion (lines 238-241) the authors discuss the potential importance of photosynthetic activity in the cotyledons. It would be helpful if they included a discussion about how the practice of using shade cloth during graft recovery may interact/interfere with this model. I also noticed that the authors did not block the light during graft recovery in their experiments, which is a common practice in grafting. I think it would be helpful to discuss how that may have influenced the timing of junction formation.
In the materials and methods section, the authors need to add more detail regarding their confocal imaging experiments. The laser settings, power, PMT info are not listed.
The transport assays for Figures 1-3 should include supplemental images for each of the time points that were quantified, and more detail about how mobile fluorescent signal versus autofluorescence were detected.
Supplemental Figure S4 would be nice to include as a main figure in the Discussion.
This is a small detail, but the authors misuse the term “graft chimera” in multiple places in the manuscript. A graft chimera is a chimeric shoot that arises from the graft junction. I would recommend just using the term “grafted plant” to avoid confusion.

First revision

Author response to reviewers' comments

Response to reviewer comments
- Comments and questions from reviewers
- Response by authors

We thank the three reviewers for taking time to review our manuscript and appreciate their comments which have improved the manuscript. Our specific points are below.

Reviewer 1

Comments for the Author:

However, I have some comments and questions to the Authors:

1. Experiments were performed with tomato and Arabidopsis seedlings. The Authors observed that increasing the recovery temperature from 27ºC to 30ºC did not promote Arabidopsis graft formation, suggesting that 27ºC is close to the maximum thermo-induction effect (lines 123-125, Results), whereas in experiments with tomatoes observed that grafted seedlings recovered at 30ºC showed significantly faster and higher phloem connection rates (lines 117-118, results). In turn, in Arabidopsis pSUC::GFP and DOF6::Venus lines cambium formation and phloem connection rate was similar to tomato grafting. How could it be understand? Could I ask about short comment to your observations? I think it should be clarified and precisely described in Results.

Response - Indeed, we see similar rates of reconnection between tomato and Arabidopsis though phloem reconnects approximately one day earlier in Arabidopsis (Figure 1). Previous studies have shown that Arabidopsis seedlings form phloem connections at 3 DAG and xylem connections at 6 DAG (Yin et al., 2012, Melnyk et al 2015). Tomato plants typically take longer to form successful grafts, between 4-6 DAG for phloem and xylem (Cui et al 2021; with three week old plants). Our results are consistent with these previous observations. Both Arabidopsis and tomato respond to 30ºC and we did not test the maximum or minimum temperature for tomato grafting since our study used Arabidopsis to go into further mechanistic details. We have updated the text to explain the differences in reconnection rates and to cite the relevant papers (Yin, Melnyk and Cui).

2. Cambium, the lateral meristem responsible for the vascular development plays important role during the vasculature healing after grafting or wounding. Is it possible to include some microscope images showing the cambium response (i.e. higher/lower cambial cell divisions) on the elevated temperature induction? It would enrich very much the observed changes during vascular regeneration in grafted seedlings and results presented in the manuscript. I will be very satisfied you supplement your results with cambium reaction.

© 2022. Published by The Company of Biologists under the terms of the Creative Commons Attribution License (https://creativecommons.org/licenses/by/4.0/).

Response - We thank the reviewer for this helpful suggestion. We have performed confocal imaging of the graft and imaged and quantified the vascular bundle during elevated temperatures. We present these new data in Figure 1G,H. We observed that high temperatures accelerated and enhanced the size of the regenerating vascular bundle providing us with additional evidence that elevated temperatures induce vascular regeneration.

3. Fig. 2D shows GUS staining of pYUCCA8::GUS Arabidopsis line, grown at 20ºC and 27ºC for 48h. GUS reaction has not been observed on the image with the seedling grown at 20ºC. I expect poor signal, but not lack of the YUC8 expression. Could the Authors comment these results?

Response - We have indeed observed some signals from *pYUC8::GUS* seedlings treated with 20ºC for 48h, however the signals were very faint and only present in the minority of the tested plants (i.e. 2/22, figure below, number at the bottom right represents the proportion of the shown individuals. Scale bars=1 mm.). We are therefore showing the images that most represent the tested samples.

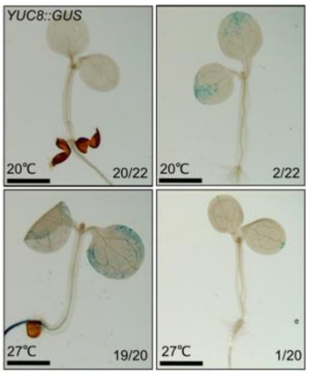

4. Slight deficiency of the manuscript is unclear experimental establishing in some point of the described methodology. Following questions:

4.1. How old seedlings were used for the analyses? I have found in the Material and methods that: ‘for three-segment cotyledon-hypocotyl grafting, cotyledon grafting was first performed when plants were four days old, then after three days of recovery at 20ºC, the attached plants were used for the hypocotyl grafting’ (lines 282-285, M&M); ‘tomato grafting was performed using seven-day-old seedlings’ (line 290, M&M); GUS reaction was analyzed in 8-day-old seedlings and for the CFDA treatment I could not found how old seedlings were used. It should be clarified in the M&M chapter.

Response - The age of plant materials have been clarified in the material and methods.

4.2. Number of analyzed plants per each of the performed experiment is confused, i.e. n=40- 80 plants per each of temperature treatment (Fig.1D) or n=30-45 plants (Fig.2A, E; Fig.3C) etc. In my opinion this is not precise and make unclear how many plants were finally analyzed per each of the experiment. I suggest to add ‘respectively’ in such record or simply assign one number of plants used/analyzed in each of experiment. Other suggestion – it should be clarified in M&M chapter.

Response - We have clarified and mentioned the sample size in the figures and/or figure captions.

5. Maybe it would be worth to discuss the temperature-induced effect on vascular tissue regeneration in grafting seedlings with other works on the vasculature healing after wounding (i.e. Sauer et al. 2006, Genes Dev. 20: 2902-2911; Balla et al. 2011, Plant J. 65: 571-577; Hajny et al. 2020, Science)?

Response - Our discussion is unfortunately quite limited due to word limit constraints to fit Development report formatting. We have removed 300 words from our previous version including half of our discussion section and our current manuscript is now closer to 3000 words. Although this would be an interesting point, we would have to remove additional discussion or results, so would prefer not to get into details regarding auxin canalization and wound healing.

© 2022. Published by The Company of Biologists under the terms of the Creative Commons Attribution License (https://creativecommons.org/licenses/by/4.0/).

Minor points

1. Line 62, 172 – dots in the middle of the sentences.

Response - We have removed the dots accordingly.

2. Should be used the first letter of the name during citation? – lines 62, 97 in the text.

Response - We have corrected the citation format accordingly.

3. Fig. 3A – lack of the ‘days after grafting’ in the graph axis description.

Response - We have added “days after grafting” in Figure 3A as suggested.

Reviewer 2

Comments for the Author:

From the general to the specific:

1. The grafting experiments heavily depend on the methodological approach of inferring vascular continuity from fluorescent dye (CFDA) or protein (GFP) diffusion. Although this analysis of functional vasculature regeneration “improved” at high temperature is certainly valid and important, the argument would be significantly strengthen by a parallel morphological analysis of the grafting zone. The authors should consider including time-lapse, high-resolution images of vasculature regeneration at low/high temperature, to correlate with the functional data already presented.

Response - We thank the reviewer for this helpful suggestion. We have performed confocal imaging of the graft junctions responding to the recovery temperatures for up to 4 days after grafting, and measured the vascular bundle size. We saw that high temperatures accelerated and enhanced the size of the regenerating vascular bundle particularly in the scion, that consisted of cambium, pericycle, phloem and xylem (Fig 1G,H), providing us with additional evidence that elevated temperatures induce vascular regeneration.

2. Related to the point above, is it possible that high temperature is simply increasing the CFDA/GFP diffusion rate through the tissue, rather than tissue regeneration? The authors should include controls at low/high temperature with not-grafted (i.e. intact) seedlings, at least for the CFDA experiments and perhaps with transgenic line expressing GFP only in the leaf phloem?

Response - This is a relevant point but it is unlikely that it is causing the effect we see for the following reason: CFDA assays are performed at room temperature regardless of the graft healing temperature, so CFDA transport dynamics should not be affected by elevated temperatures. To further address this concern, we have included controls (i.e. non-grafted plants) for both CFDA and GFP assays at low/high temperatures at different time points for phloem (Fig S1) and xylem (Fig S2) connection and observed that temperature treatments did not change the diffusion rates in the controls.

3. Regarding the expression analysis (e.g. Fig 2C or Fig S2B,C), is it possible that high temperature in these conditions increases a wide range of non-specific transcriptional activity? The authors should present data about the relative expression (high/low temperature) of some housekeeping genes, as negative controls.

Response - We thank the reviewer for the comment. We have performed a relative expression analysis on *MON1*, a temperature-stable housekeeping gene (Hong et al 2010, Plant Cell Physiol), and saw that temperatures did not affect its expression in the tested genotypes (new data included in Fig S4D). We would like to further emphasize that several of the selected auxin biosynthesis (Fig S4B) and vascular (Fig S4C) genes are not affected by temperature treatments, suggesting that not all transcriptional activity is increased by high temperatures. Similar conclusions can be drawn from analyses of temperature response whole-genome transcriptomics in previously published

© 2022. Published by The Company of Biologists under the terms of the Creative Commons Attribution License (https://creativecommons.org/licenses/by/4.0/).

studies (e.g., Bellstaedt et al., 2019, Plant Phys).

4. The error in the estimate of a proportion from a sample can be quantified with a “standard error of the proportion”, or s.e.p.). Although the statistical test when comparing proportions used by the authors seems appropriate and their conclusions correct, they should add error bars with s.e.p. in all their graphs reporting estimated proportions.

Response - We agree and have added s.e.p. to the proportion comparisons.

Reviewer 3

Advance Summary and Potential Significance to Field:

In this manuscript by Serivichyaswat et al., the authors connect the phenomenon of heat-enhanced graft formation with molecular regulators for auxin biosynthesis, response, and temperature perception. The authors use refined cotyledon grafting to demonstrate that auxin-dependent temperature perception and response occurs in distant leaf tissues, rather than locally at the graft interface. Furthermore, they extend their model beyond grafting, by demonstrating that plant parasite haustorium formation is also enhanced by elevated temperatures. Overall, this paper is relevant to work on regeneration biology and agricultural research on grafting, and thus of broad interest to the research community. The experimental methods are generally robust, although I list some suggestions that I think would improve the study below, and I’m particularly impressed by the cotyledon grafting.

Comments for the Author:

Successful graft formation in this study is based on resumed physiological xylem and phloem transport. I would like to see this paired with some detailed anatomical verification of vascular connections within the graft junction.

Response - We thank the reviewer for this helpful suggestion. We have now included anatomical details regarding graft junction morphology over a time course (Fig 1G,H). We feel this is a very useful addition and strengthens the manuscript.

In the discussion (lines 238-241) the authors discuss the potential importance of photosynthetic activity in the cotyledons. It would be helpful if they included a discussion about how the practice of using shade cloth during graft recovery may interact/interfere with this model. I also noticed that the authors did not block the light during graft recovery in their experiments, which is a common practice in grafting. I think it would be helpful to discuss how that may have influenced the timing of junction formation.

Response - This is a good point, but unfortunately due to the word count limitations, we have now shortened the discussion and removed this point. Regarding blocking light during graft recovery, we have previously tested this aspect and found that darkness negatively affected graft formation as we did not see any phloem reconnection for up to 7 days after grafting. Previous studies have found that low light levels benefitted grafting (ie Bartusch et al 2020, Plant Methods) but we did not investigate this aspect further and instead focused on temperature.

In the materials and methods section, the authors need to add more detail regarding their confocal imaging experiments. The laser settings, power, PMT info are not listed.

Response - We have added the laser settings to the materials and methods section (i.e. Calcoflor White staining: 405 nm excitation, 2% laser power, 410-529 nm detection, and 210 PMT. GFP and mVenus: 488 nm excitation, 10% laser power, 500-524 nm detection, and 280 PMT.).

The transport assays for Figures 1-3 should include supplemental images for each of the time points that were quantified, and more detail about how mobile fluorescent signal versus autofluorescence were detected.

© 2022. Published by The Company of Biologists under the terms of the Creative Commons Attribution License (https://creativecommons.org/licenses/by/4.0/).

Response - We have included in the supplemental section images of each time point, showing the signals in root tips and/or shoots as well as the non-grafted controls (Fig S4, S5).

Supplemental Figure S4 would be nice to include as a main figure in the Discussion.

Response - We have moved Fig S4 to Fig 4.

This is a small detail, but the authors misuse the term “graft chimera” in multiple places in the manuscript. A graft chimera is a chimeric shoot that arises from the graft junction. I would recommend just using the term “grafted plant” to avoid confusion.

Response - We agree and have corrected the terminology accordingly.

Second decision letter

MS ID#: DEVELOP/2021/200079

MS TITLE: High temperature perception in leaves promotes vascular regeneration and graft formation in distant tissues

AUTHORS: Phanu T Serivichyaswat, Kai Bartusch, Martina Leso, Constance Musseau, Akira Iwase, Yu Chen, Keiko Sugimoto, Marcel Quint, and Charles W. Melnyk
ARTICLE TYPE: Research Report

I am happy to tell you that your manuscript has been accepted for publication in Development, pending our standard ethics checks.

Reviewer 1

Advance summary and potential significance to field

Dear Authors,

I would like to thank you for consider my suggestions and prepare the final version of the manuscript with appropriate accuracy.

Comments for the author

The improved version of the manuscript is now enough clear and satisfactory corrected. The significant points such as additional clarifications for the applied methodology and obtained results were made.
I recommend this manuscript for publication.

Reviewer 2

Advance summary and potential significance to field

This work describes the effects of temperature perception onto vasculature regeneration during grafting. The fact that auxin signalling is involved in the process is perhaps not suprising, but it's important to have experimental evidence of that. These results are certainly of interest to developmental biologists focused on regeneration processes, and it has potential for future applications in the agritech sector.

© 2022. Published by The Company of Biologists under the terms of the Creative Commons Attribution License (https://creativecommons.org/licenses/by/4.0/).

Comments for the author

The authors fully addressed my previous concerns and produced clear new images that enhance their work.
I recommend the publication of the manuscript in this revised form.

Reviewer 3

Advance summary and potential significance to field

This paper is relevant to work on regeneration biology and agricultural research on grafting, and thus of broad interest to the research community. The experimental methods are generally robust, although I have some additional suggestions that are quite minor.

Comments for the author

The authors have addressed all of the issues raised during the first round of review. I have two additional requests and one question.
First, the added anatomical imaging and vascular quantification in figure 1 G-H is a nice addition, however, the authors should annotate the images in 1G so that it's clear how vasculature was quantified.
Second, the supplemental images supporting the CFDA and GFP transport assays are nice, but I think the paper would be stronger if the authors included images for all of the replicates that were used to quantify transport in their supporting data.
One question that I have after seeing Fig 1 G, is that the rootstock-scion are physically disconnected at 3 DAG (@20 deg) and at 2 DAG (@27 deg), but the transprort assays show ~20% phloem connectivity. Can the authors explain this difference between anatomical restoration and physiological transport?

© 2022. Published by The Company of Biologists under the terms of the Creative Commons Attribution License (https://creativecommons.org/licenses/by/4.0/).
